# Cultural and socioeconomic determinants of family satisfaction with ICU care across the globe: a scoping review

**DOI:** 10.3389/fmed.2025.1700383

**Published:** 2026-01-15

**Authors:** Margiben Tusharbhai Bhatt, Sunil Ravindranath, Souvik Chaudhuri, Shreya Nair, Anuja Damani, Bhumika Tumkur Venkatesh, Sujata Shirodkar

**Affiliations:** 1 Department of Critical Care Medicine, Kasturba Medical College, Manipal Academy of Higher Education, Manipal, India; 2 Department of Palliative Medicine and Supportive Care, Kasturba Medical College, Manipal Academy of Higher Education, Manipal, India; 3 Campbell South Asia, New Delhi, India

**Keywords:** caregivers, communication, culture, educational status, family, healthcare, intensive care units, palliative care

## Abstract

**Objective:**

To map evidence on global cultural and socioeconomic determinants of family satisfaction with care in the intensive care unit (ICU), including assessment, the influence of cultural beliefs and practices, and the impact of socioeconomic status on family satisfaction.

**Study design:**

This scoping review followed the Joanna Briggs Institute (JBI) guidelines and was reported in accordance with PRISMA-ScR. A comprehensive search of English-language studies was conducted across multiple databases. Studies were included if they examined the impact of cultural and socioeconomic factors on shared or end-of-life decision-making among family members, or if they explored the relationship between these factors and psychological outcomes (e.g., anxiety, depression, insomnia) in caregivers during ICU stays—provided that family satisfaction was also assessed. Two independent reviewers screened all studies, and data were extracted using a customized form to ensure consistency and relevance to the research question.

**Results:**

A total of 2,121 articles were identified from PubMed (*n* = 68), Scopus (*n* = 475), Embase (*n* = 1,436), Web of Science (*n* = 138), Cochrane (*n* = 4), and other sources (*n* = 10). After removing duplicates, 1,772 articles underwent title and abstract screening, with 101 full texts evaluated. Seventeen studies met the inclusion criteria. Data extraction focused on family characteristics (e.g., religion, race, education, kinship, prior ICU experience), sociocultural and economic factors, end-of-life practices, and ICU-related elements influencing family satisfaction. Findings were narratively synthesized to provide a comprehensive overview of these contextual influences. The review revealed that prioritizing patient symptoms, fostering effective communication to support shared decision-making, and showing compassion toward family needs significantly enhanced family satisfaction. However, a major gap was identified in research from low- and middle-income countries (LMICs). Further studies are needed to understand how diverse cultural and socioeconomic factors affect caregiver satisfaction in these settings and to develop tailored strategies that improve family experiences and outcomes in intensive care units globally.

**Conclusion:**

This review comprehensively mapped the available evidence and noted that addressing the cultural, socioeconomic, religious, and spiritual needs of the family, including comfort-based care interventions in the ICU, may improve family perceptions of ICU care and satisfaction. Further studies are required, especially in LMIC settings, to address diverse races, religions, and ethnicities in this context.

**Systematic review registration:**

https://osf.io/5xusk.

## 1 Introduction

Family satisfaction has emerged as a critical metric in evaluating the quality of care provided in ICUs. Families of severely ill patients often act as “surrogate decision-makers” and are central to patient-centered care models. Their experiences, perceptions, and satisfaction levels can significantly impact both the quality of care provided and their psychological outcomes, including the risk of anxiety, depression, and post-traumatic stress disorder after an ICU stay (1, 2).

Cultural factors profoundly shape family expectations, preferences, and satisfaction with ICU care. Variations in beliefs about illness, death, and medical decision-making across cultures influence communication styles, preferences for “shared decision-making,” and perceptions of empathetic care (3, 4). Religious and spiritual practices often intersect with palliative care, further influencing how families perceive the adequacy of emotional and spiritual support provided by healthcare teams (5).

Socioeconomic factors, including income, education, and occupational status, also play a pivotal role. These determinants influence how families interact with ICU processes, comprehend medical information, and assess the quality of care. Lower socioeconomic status has been linked to limited health literacy, poorer healthcare experiences, and feelings of exclusion from critical decision-making (6). In resource-limited or culturally diverse settings, these disparities can further hinder effective communication and erode trust in healthcare providers (7).

Considering the “global commitment to Universal Health Coverage and Sustainable Development Goal 3.8 (access to quality essential health services),” there is growing recognition of the need to understand sociocultural determinants in ICU care (8). An in-depth understanding of how cultural and socioeconomic aspects influence family satisfaction is crucial for developing equitable, person-centered critical care practices. Mapping the existing evidence in this area can identify priority areas for culturally sensitive interventions and inform policies to reduce inequities in ICU experiences.

Scoping reviews offer a robust method for addressing these complex, context-dependent questions. By systematically identifying, mapping, and synthesizing existing research, this review provides a detailed overview of the existing evidence base. It also highlights key gaps, guiding future research and informing ICU practices that are responsive to diverse cultural and socioeconomic needs.

## 2 Material and methodology

This scoping review adhered to the methodological framework established by the Joanna Briggs Institute (JBI) for conducting scoping reviews (9). The final report was prepared in accordance with the PRISMA-ScR (Preferred Reporting Items for Systematic Reviews and Meta-Analyses extension for Scoping Reviews) checklist (10). Owing to the broad and diverse nature of the literature on communication between patients, families, and caregivers, a scoping review was considered the most suitable method for capturing and synthesizing evidence across diverse study types and international contexts. Unique Identifier: https://osf.io/5xusk (registered with OSF on 08.07.2025) URL of publicly accessible website: https://osf.io/. A formal protocol was not published for this review (Appendix 1).

### 2.1 Eligibility criteria

#### 2.1.1 Data sources and search strategy

An extensive literature search was performed to gather studies examining the cultural and socioeconomic determinants of family satisfaction with ICU care across global settings. The search strategy was designed to capture diverse study methodologies—including qualitative, quantitative, and mixed-methods research—across diverse cultural and socioeconomic contexts.

The databases searched included PubMed/MEDLINE, Embase, the Cochrane Library, Scopus, and Web of Science. All search strategies included both controlled vocabulary (e.g., MeSH terms in PubMed, Emtree in Embase) and relevant free-text terms to ensure comprehensive coverage and sensitivity (see Table 1).

**Table 1:** Search strategy according to the Population–Concept–Context (PCC) framework, with inclusion and exclusion criteria.

Framework	Inclusion criteria	Exclusion criteria
Population	Studies that examine cultural and socioeconomic factors influencing family members’ or patient caregivers’ satisfaction with intensive care in adult ICU settings worldwide.	Studies focus only on healthcare interprofessional communication and do not involve patient or family satisfaction.
Concept	Studies that explore the impact of sociocultural and economic factors on shared decision-making by family members, where family satisfaction is reported as an outcome. Studies examine how sociocultural and economic factors influence palliative care decision-making involving family caregivers, with family satisfaction included as an outcome measure. Studies that investigate the relationship between sociocultural and economic factors and psychological outcomes (such as anxiety, depression, and insomnia) in patients’ families during their ICU admission, provided family satisfaction is also assessed.	Studies that examine sociocultural or economic influences on family decision-making but do not measure or report family satisfaction. Studies that focus solely on the psychological impact (e.g., anxiety, depression, insomnia) on family members, and studies addressing shared or end-of-life decision-making without linking these processes to family satisfaction.
Context	Studies conducted in various adult ICU settings across different global regions and cultural contexts, focusing on communication strategies and their impact on patient, family member, or family satisfaction.	Studies that report on communication practices occurring solely in non-clinical areas of healthcare settings (e.g., administrative or community-based environments) do not focus on the ICU context.
Type of evidence resources	Studies published in the English language, peer-reviewed, with full-text articles available, including both qualitative and quantitative research designs. Experimental and quasi-experimental studies, comprising randomized controlled trials (RCTs) and quasi-RCTs, as well as observational studies, including prospective and retrospective cohort studies, case–control studies, analytical cross-sectional studies, descriptive observational designs, and descriptive cross-sectional studies.	Studies reported in non-English languages; review articles of any kind, including systematic reviews, narrative reviews, scoping reviews, and other secondary review types; documentaries; non-scientific case studies; non-peer-reviewed media sources; and grey literature, including theses, dissertations, and book chapters.

Search terms.

were organized around four core conceptual domains, consistent with the Population–Concept–Context (PCC) framework:

1. ICU setting (e.g., “intensive care units,” “critical care,” “ICU”)
2. Cultural factors (e.g., “culture,” “cross-cultural,” “ethnicity,” “religion”)
3. Socioeconomic factors (e.g., “income,” “education,” “socioeconomic status,” “employment”)
4. Family satisfaction (e.g., “family satisfaction,” “patient satisfaction,” “family experience,” “FS-ICU 24R”).

Boolean operators (AND, OR) and appropriate truncation were applied to integrate and expand search terminologies across the four domains. The initial search technique was developed for PubMed and subsequently adapted for each additional database, accounting for differences in syntax and controlled vocabulary. The complete database-specific search strategies are provided in Appendix 2.

#### 2.1.2 Data extraction and management

Search results across databases and additional records from other sources were transferred to Rayyan (11), and duplicates were removed.

#### 2.1.3 Study selection

Two reviewers independently selected studies through a two-stage screening process.

**Figure 1:**
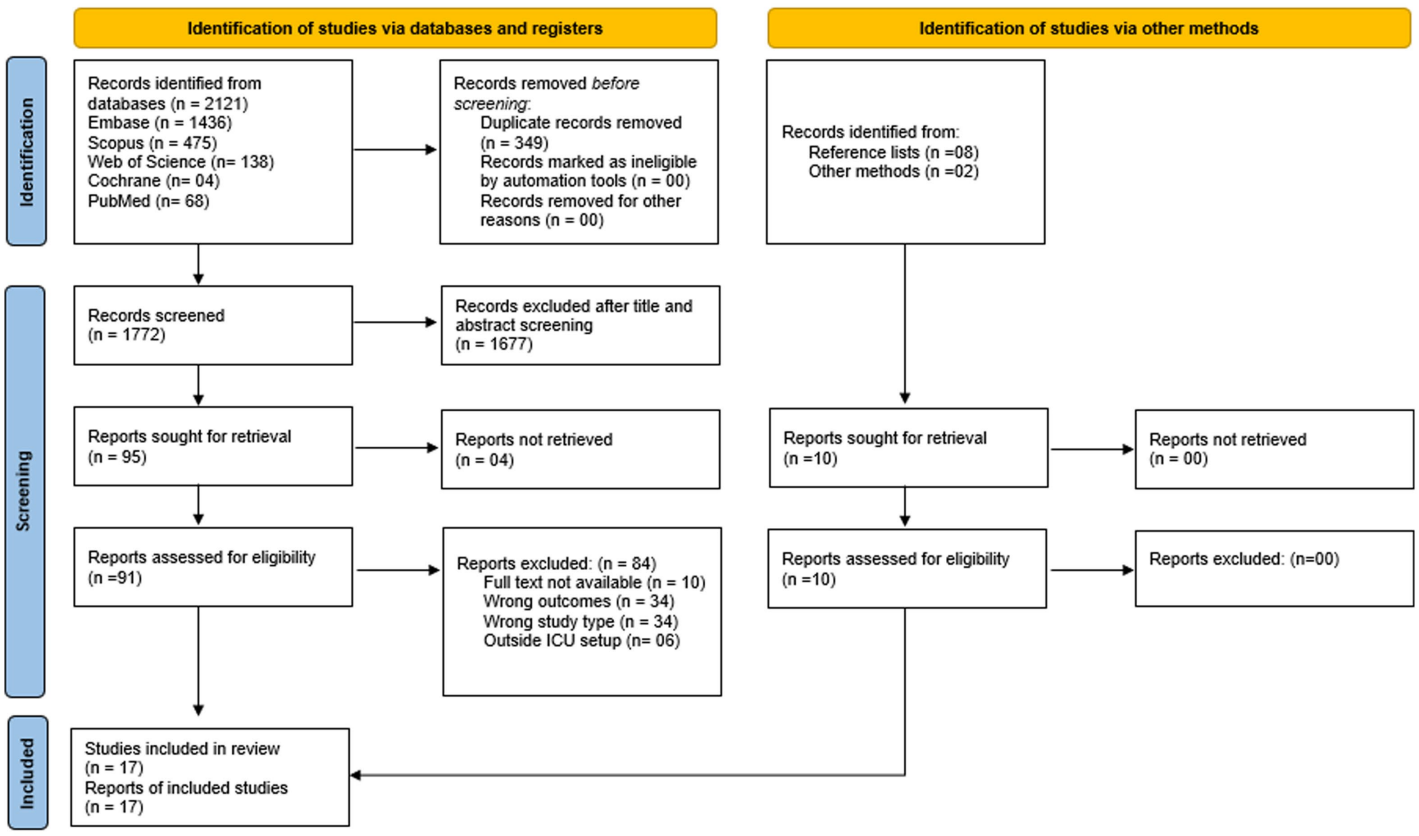
PRISMA flow diagram. Source: Page MJ, et al. BMJ 2021;372:n71. doi: 10.1136/bmj.n71.

During the first stage, two reviewers screened titles and abstracts to determine relevance according to predefined inclusion criteria. Studies that appeared eligible were advanced to the second stage; any disagreement between the two reviewers was resolved through a consensus-based discussion. Studies that appeared eligible advanced to the next stage, where full-text articles were reviewed by two reviewers to determine suitability for inclusion. Articles meeting the inclusion criteria were then subjected to a final eligibility check to ensure conformity with the predefined framework. Any conflicts between the two reviewers concerning study eligibility were addressed through consensus with a third reviewer. All disagreements were resolved through discussion. The primary reasons for excluding full-text articles were documented in the PRISMA 2020 flow diagram (Figure 1).

#### 2.1.4 Data charting

A pretested coding framework was employed, including detailed variables such as citation details, study objectives, research design, country, setting, population characteristics, cultural and socioeconomic factors, family satisfaction measures or other tools, and reported outcomes. Data charting was carried out independently by two reviewers (Tables 2, 3).

**Table 2:** Study characteristics of the included studies.

Author, year, and country	Study design	Study objectives	Study setting	Sample size n (male/female)	Sample characteristics
Al Mutair et al. (2018) (25), Saudi Arabia	Phenomenological study	“To identify family needs, beliefs, and practices during the end-of-life care”	Tertiary referral hospital, 36 ICU beds	10 (8/2)	Mean age of 37 years. All first-degree relatives, Muslim, Arabic-speaking, literate
Amass et al. (2020) (12), USA and Italy	Multicenter, multinational, prospective before-and-after intervention trial	“Assess whether ‘Family Care Rituals’ (FCR), an informational intervention, reduces symptoms of PTSD and improves psychological outcomes and satisfaction among family members”	Three academic ICUs (two in the USA, one in Italy), medical and medical/surgical units	260 (135/125)	Mean age ~ 64 years; majority white race (75% usual care, 81% intervention); ~20% Italian; ~46–48% with college degree
Atri et al. (2024) (13), India	Prospective, single-center, questionnaire-based observational study	“To Identify factors affecting family satisfaction”	Multidisciplinary, tertiary care, academic hospital, 30-bed ICU	336 (215/121)	Mean age ~56.5 years; 72.3% immediate kin; 88% no prior ICU experience; 51.9% educated to pre-university or lower; majority rural background; 32.4% out-of-pocket expenditure
Bhatt et al. (2024) (14), India	Prospective, single-center, questionnaire-based observational study	“To determine the effect of shared decision-making factors on family satisfaction”	Multidisciplinary ICU, tertiary care medical college hospital, 30 beds, semi-closed model	336 (215/121)	Mean age ~ 56.5 years; 72.3% immediate kin; 70.8% cohabiting with patient; 51.9% educated up to pre-university; 59.2% rural; 32.4% out-of-pocket expenditure
Chuang et al. (2016) (15), USA	Observational, cross-sectional study. Survey-based, in-person administration (with a few by phone) during ICU admission	“To evaluate differences in satisfaction with communication in the ICU between caregivers of different racial and ethnic groups”	Three ICUs in two hospitals within a major academic medical center	100 (30/70)	Mean age: 52.5 years; 69% immediate kin; 66% had previous ICU experience; 100% with medical insurance; Black, non-Hispanic 34.45, Hispanic/Latino 31.3%, white, non-Hispanic 18.8%
Curtis et al. (2011) (16), USA	A cluster-randomized trial with intervention	“To evaluate effectiveness of quality improvement intervention to improve ICU end-of-life care”	15 hospitals (three pilot, six in the intervention group, and six in the control group)	822 (130/692)	Mean age ~ 60 years; majority white ~84–89%; ~43–48% spouse
Fumis et al. (2015) (17), Brazil	Prospective, single-center, questionnaire-based observational study	“To assess satisfaction, anxiety, and depression symptoms in family members of ICU patients in an open visit ICU”	22-bed mixed medical-surgical ICU, tertiary private teaching hospital, 24-h open visiting policy	471 (103/368)	Median age ~ 54 years; 79% with higher education; 90% immediate kin; 69% Catholic; 73% with prior ICU experience
Gerstel et al. (2008) (18), USA	Multicenter cohort study. Prospective questionnaire-based survey with retrospective retrieval of the records	“To evaluate withdrawal of life support and its influence on family members’ overall satisfaction with care”	14 hospitals, including both university-affiliated and community hospitals	584	90.6% white, 97.5% with medical insurance, 93.32% more than two decision-makers
Hajj et al. (2015) (19), Lebanon	Cross-sectional descriptive study	“What is the reliability and validity of the CCFSS in a Lebanese population? How satisfied are family members, and what factors affect family satisfaction?”	Advanced academic medical center hospital with 29-bed ICU	123 (44/79)	Mean age ~ 43.7 years; 86.2% secondary education or above; 78% immediate kin; 67.5% were living close to hospital; 47.9% with prior ICU experience;79.8% Muslim
Heyland et al. (2002) (26), Canada	Multicenter prospective survey	“To determine levels of family satisfaction in ICU”	Six tertiary care hospitals affiliated with medical schools, both medical and surgical ICUs, with bed strengths varying from 8 to 24	620 (190/430)	Mean age 52.2 years; 92% white; female majority (69%); 84% immediate kin; 60% cohabitating with patient; 39% living close to hospital; 44% post-secondary education;49% satisfied with previous ICU experience
Johnson et al. (2014) (20), USA	“Before after” trial evaluating the effect of multidisciplinary quality improvement intervention to improve palliative and end-of-life care in ICU	“To evaluate the associations between spiritual care providers’ activities and family ratings of satisfaction with care”	Tertiary care teaching and Level I trauma center hospital with a 65-bed ICU	275 (96/179)	81.9% non-Hispanic white, mean age 56.4 years; 82.1% immediate kin; 57.7% cohabitating with patient
Jones et al. (2018) (21), USA	Secondary analysis of an exploratory descriptive design	“To describe family members’ experience with bereavement in ICU”	32-bed medical-surgical and 16-bed cardiac ICU of a tertiary care hospital	17 (5/12)	Mean age 62.4%; 88% immediate kin, 82% white; 94% college graduate; 47% prior ICU experience; 82% cohabitating with patients; 47% documented family conference, palliative consult, or advance directive
Kaufer et al. (2008) (28), USA	Prospective, single-center, questionnaire-based observational study	“To examine family satisfaction with end-of-life care before and after a palliative care intervention”	Medical ICU, urban inner-city hospital	88	67% of the family members were Black
Midega et al. (2019) (22), Brazil	Questionnaire-based longitudinal observational study	“To analyse the satisfaction with ICU support and symptoms of anxiety/depression in family members”	20-bed ICU in a public general hospital	3 (09/26)	Mean age 43.2 years; 21% married; 31% belonging to the Catholic/Evangelical religion; 11% with higher education
Min et al. (2018) (23), South Korea	Prospective, multicenter, observational survey study	“To evaluate family satisfaction with ICU care and decision-making”	Medical, Surgical, and emergency ICUs in three tertiary teaching hospitals	200 (100/100)	Mean age 51.6 years; immediate kin 88.5%; prior experience with ICU 38%; cohabitation with patient 52.5%
Nayfeh et al. (2021) (27), Canada	Prospective, single-center, questionnaire-based observational study	“To evaluate family satisfaction with the quality of end-of-life care”	Large urban academic tertiary care hospital	1,543	Immediate kin 88%; majorly White (68.2%), followed by 10.5% Mediterranean; majorly Christian (66.6%), Jewish (12.3%), Muslim, others smaller proportions, 58.6% very spiritual
Schwarzkopf et al. (2013) (24), Germany	Prospective cohort study	“To assess family satisfaction in ICU. To identify areas for improvement in ICU care based on family experience”	Four adult ICUs (two mixed surgical ICUs, one medical ICU, one neurological ICU), a total of 72 ICU beds	213 (62/151)	Majority 45 + years; immediate kin 95.3%

**Table 3:** Factors assessed, tools used, and key findings from the included studies.

Author, year, country	Cultural and socioeconomic factors assessed	Family satisfaction tools/any other tools used	Key findings	Conclusion
Al Mutair et al. (2018) (25), Saudi Arabia	Cultural factors: religious practices, interpretation of death as God’s will, importance of spiritual care, and need for an ICU environment to accommodate large families. Socioeconomic factors: not explicitly assessed	In-depth interviews	Family’s experience during end-of-life care reflected in four major themes: (a) spirituality of death, (b) family’s need for information, (c) being there, and (d) the ICU environment.	Introduction of a model of care that delivers a holistic approach: accommodating the family’s spiritual needs, frequent communication with the healthcare team, relaxed visitation policy, and a family-friendly ICU environment
Amass et al. (2020) (12), USA and Italy	Cultural factors: rituals and traditions around death, family racial/ethnic composition. Socioeconomic factors: education level, relationship to the patient	FS-ICU 24 questionnaire at 90 days post-ICU discharge/death. HADS and IES-R scale for post-traumatic stress disorder (PTSD)	Primary outcome: PTSD symptoms in family members 90 days after patient death or ICU discharge. Secondary outcomes: depression, anxiety, and family satisfaction. Findings: PTSD symptoms were significantly lower postintervention vs. preintervention (27.1% vs. 39.2%; OR 0.58, *p* = 0.046). No significant differences were observed for depression (26.5% vs. 25.2%; OR 0.93, *p* = 0.818), anxiety (41.0% vs. 45.5%; OR 1.20, *p* = 0.234), or satisfaction scores (89.0 vs. 85.1; OR 3.85, *p* = 0.052).	Family care rituals may reduce PTSD symptoms in ICU families
Atri et al. (2024) (13), India	Cultural factors: family as surrogate decision-maker, values of beneficence and end-of-life ethics, family involvement in decision-making, perception of the ICU environment, and appraisal of multidisciplinary counseling. Socioeconomic factors: education/literacy status, urban vs. rural residence, and financial coverage.	Family satisfaction in the ICU-24 revised (FS-ICU 24R)	Factors associated with family satisfaction: treatment of patients’ physical symptoms (OR 3.73, *p* = 0.003), ICU staff’s consideration of family needs (OR 4.44, *p* < 0.001), and concern/care for family (OR 2.67, *p* = 0.023). Not associated with satisfaction: patient survival (*p* = 0.331), ICU stay (*p* = 0.328), or hospital stay (*p* = 0.865).	Emotional support, a favorable ICU/waiting room environment, consideration of family needs, and effective symptom management are key to family satisfaction
Bhatt et al. (2025) (14), India	Cultural factors: family structure and kinship roles, prior ICU experience, multiple decision-makers, cultural emphasis on physician-led decisions. Socioeconomic factors: education level, source of healthcare financing, rural vs. urban residence.	FS-ICU 24R	Factors associated with family satisfaction: consistency of information (OR 8.71, *p* < 0.001), honesty of information (OR 7.04, *p* < 0.001), and frequency of doctor–family communication (OR 6.25, *p* < 0.001). Not associated with satisfaction: number of decision-makers (*p* = 0.463) or prior ICU experience (*p* = 0.430).	Frequent doctor–family communication with honest and consistent information, along with shared decision-making, is vital for family satisfaction
Chuang et al. (2020) (15), USA	Cultural factors: patient and caregiver self-identified race/ethnicity. Socioeconomic factors: insurance status, education level, residence, and cohabitation with the patient.	FS-ICU 24R and QOC questionnaires	Mean FS-ICU scores were similar across racial/ethnic groups—white 84.2 (SD 20.5), Black 83.3 (SD 16.2), Hispanic/Latino 82.7 (SD 17.8), Other 80.9 (SD 18.8) (*p* = 0.92). Differences remained non-significant after adjusting for patient and respondent characteristics.	The study was inconclusive on whether the quality of communication influences disparities in end-of-life care
Curtis et al. (2011) (16), USA	Cultural factors: patient race/ethnicity, minority status. Socioeconomic factors: Marital status, education level.	FS-ICU and QODD (family and nurse versions, validated, multi-item and single-item scores) questionnaires	Primary outcome: family-QODD showed no improvement with intervention (*p* = 0.33). Secondary outcomes: no change in family satisfaction (*p* = 0.66) or nurse-QODD (*p* = 0.81). Non-significant increase in ICU days before death (HR 0.9, *p* = 0.07). No change in time from admission to withdrawal of mechanical ventilation (HR 1.0, *p* = 0.81).	Clinician-targeted quality improvement intervention did not improve the quality of dying or family satisfaction. Interventions should instead focus on direct engagement of patients and families
Fumis et al. (2015) (17), Brazil	Cultural factors: family structure/practices (strong family presence, family-centered decision-making), Catholicism, family coping/support needs, prior ICU experience. Socioeconomic factors: education level, relationship to patient.	Modified CCFNI and HADS questionnaire	Anxiety and depression prevalence were 34 and 17%, respectively. Positive association between relaxed visiting hours and satisfaction (*p* = 0.004). Dissatisfied families reported higher rates of anxiety and depression (*p* = 0 0.001). Patients’ severity showed a trend toward dissatisfaction (*p* = 0.08) and affected emotional disorders (*p* = 0.001).	Families with relaxed ICU visitation policies reported high satisfaction and lower incidence of anxiety and depression Open visitation polices serve as a protective factor for families with higher HADS scores
Gerstel et al. 2008 (18), USA	Cultural factors: predominantly white population, role of culturally sensitive communication and spiritual support. Socioeconomic factors: education level, financial support, prior ICU experience.	FS-ICU	Among six life-sustaining interventions, ~50% of patients (271/584) had withdrawal lasting >1 day. Prolonged withdrawal was associated with younger age, longer ICU stay, more life-sustaining treatments, and involvement of more decision-makers. In longer ICU stays, extended withdrawal was linked to higher family satisfaction (*p* = 0.037), and extubation before death further increased satisfaction (*p* = 0.009).	Gradual withdrawal of life supports is associated with greater family satisfaction and should be regularly practiced. When feasible, extubation before death should be encouraged
Hajj et al. (2015) (19), Lebanon	Cultural factors: religion and need for religious support. Socioeconomic factors: education level, prior ICU experience.	Arabic version of the CCFSS questionnaire	The CCFSS demonstrated good reliability and validity in the Lebanese population. The lowest satisfaction was in the comfort domain and the highest was in assurance. Younger, educated relatives reported lower satisfaction; Christian families reported less satisfaction with informational needs. Not associated with satisfaction: sex, distance from hospital, relationship to patient, prior ICU experience, and diagnosis.	Evaluating family satisfaction across different cultures is important, as each culture has unique needs that must be understood
Heyland et al. (2002) (26), Canada	Cultural factors: not explicitly assessed. Socioeconomic factors: education level.	Modified version of PJHQ questionnaire	Families reported the highest satisfaction with nursing skill/competence (92.4 ± 14.0), compassion and respect for the patient (91.8 ± 15.4), and pain management (89.1 ± 16.7). The lowest satisfaction was reported for the waiting room atmosphere (65.0 ± 30.6) and the frequency of doctor communication (70.7 ± 29.0).	Enhancing the quality of interactions and communication with families is likely to further increase satisfaction
Johnson et al. (2014) (20), USA	Cultural factors: addressal of cultural needs, intrafamily disagreements. Socioeconomic factors: education level.	FS-ICU	Discussions about the patient’s wishes for end-of-life care and a greater number of spiritual care activities performed were associated with increased overall family satisfaction with ICU care (*p* < 0.05).	Findings provide insight into the role of spiritual care providers and into addressing spiritual needs in the ICU associated with high family satisfaction
Jones et al. (2018) (21), USA	Cultural factors: visit from pastor, race/ethnicity. Socioeconomic factors: education level.	FS-ICU, with qualitative open-ended questions	Three major themes emerged: (1) bereavement was an individual experience; (2) events during ICU stay had a lasting impact on families even after a year of death; (3) social, cultural, spiritual, and religious events following a patient’s death are significant for families of ICU patients.	Bereavement is a difficult experience for the families of patients who died in the ICU. Targeted interventions to support bereaved families can increase overall family satisfaction
Kaufer et al. (2008) (28), USA	Cultural factors: high proportion of Black/minority patients, language barriers, cultural implications for palliative care. Socioeconomic factors: no formal income/employment measures reported.	FS-ICU	Compared to preintervention, the intervention significantly improved family satisfaction with decision-making, communication with physicians and nurses, and the death and dying process.	Early, structured communication and support can meaningfully improve family satisfaction with ICU care Culturally responsive interventions are essential to address diverse family needs and expectations
Midega et al. (2019) (22), Brazil	Cultural factors: religion, relationship to patient. Socioeconomic factors: education level, marital status, and prior ICU experience.	CCFNI modified and validated version of HADS questionnaire	77.1% of families were satisfied with treatment in the ICU; clear and complete information during counseling and easy accessibility to doctors were significantly correlated to overall family satisfaction. There was a high prevalence of anxiety (60%) and depression (54.3%) in family members.	Clear, complete information and frequent communication from the healthcare team lead to greater satisfaction Psychological support is paramount for emotionally distressed families
Min et al. (2018) (23), South Korea	Cultural factors: Confucian influence on communication, family-centered decision-making, and cultural taboos around death. Socioeconomic factors: hospital context in a lower socioeconomic area, relationship to the patient.	Korean translated and validated version of the FS-ICU 24 questionnaire	Satisfaction with information/decision-making was higher than satisfaction with care (78.2 ± 18.2 vs. 73.5 ± 19.4; *p* = 0.001). Families who consented to do-not-resuscitate orders and those whose patients died in the ICU reported lower satisfaction. Lowest satisfaction was noted for the ICU/waiting room environment.	Quality improvement initiatives such as clear communication, sensitive handling of end-of-life decisions, and improvements in the ICU environment are essential to enhancing family satisfaction
Nayfeh et al. (2021) (27), Canada	Cultural factors: patient race/ethnicity, religion, spirituality, preferred language, language barriers. Socioeconomic factors: no direct data on income, education, or immigration status.	EOLS Survey, modified from NRC Hospice Survey	Satisfaction was higher for patients dying in the ICU than in other locations (RR 1.51, 95% CI 1.05–2.19, *p* = 0.028). Lower satisfaction was observed among families with language/communication barriers (RR 0.49, 95% CI 0.23–1.06, *p* = 0.069) and for Muslim patients compared with other religious affiliations (RR 0.46, 95% CI 0.21–1.02, *p* = 0.056).	Communication and information sharing, illness management, and healthcare provider traits like emotional support, doctor accessibility, and time spent with patients and their families are priority areas for improving satisfaction
Schwarzkopf et al. (2013) (24), Germany	Cultural factors: qualitative themes included respect and compassion, privacy in communication, waiting room environment, and consistency of staff interactions. Differences in nurse vs. physician communication (honesty, completeness). Socioeconomic factors: no direct assessment of income, occupation, or other socioeconomic status.	FS-ICU 24R and qualitative assessment of satisfaction themes (communication, respect, emotional support, ICU environment)	High (FS-ICU total 78.3 ± 14.3/100); physicians were rated higher than nurses for honesty (*p* = 0.033) and completeness (*p* = 0.004) of information; satisfaction correlated with comments (positive r = 0.26, negative r = −0.46, both significant). Communication gaps (jargon, delays, contradictions, privacy issues) and abrupt transfers to wards negatively affected family satisfaction. Negative experiences were reported in relation to noisy and overcrowded waiting rooms.	Quality improvement in the following aspects of care: patient agitation management; consistency, clarity, and completeness of information; emotional support, respect, and compassion toward families; better amenities for visiting relatives; and a better waiting room atmosphere

#### 2.1.5 Critical appraisal

As this was a scoping review, no formal quality assessment of the studies included was undertaken, in line with JBI and PRISMA-ScR recommendations.

#### 2.1.6 Synthesis of results

Charted data were organized in tables and summarized using descriptive statistics and narrative synthesis. Data were grouped by key study characteristics and themes to map the breadth of evidence and highlight the patterns and gaps across the literature.

## 3 Results

Data presentation and interpretation were organized under the following subheadings (Table 4).

**Table 4:** Summary of findings from the included studies.

Study characteristics	Number of studies (*N* = 17)	Percentage contribution
Country		
High-income countries		
Canada	2 [Heyland et al. (26); Nayfeh et al. (27)]	11.8%
Germany	1 [Schwarzkopf et al. (24)]	5.9%
Saudi Arabia	1 [Al Mutair et al. (25)]	5.9%
South Korea	1 [Min et al. (23)]	5.9%
USA, Italy	7 [Amass et al. (12) (USA and Italy); Chuang et al. (15); Curtis et al. (16); Gerstel et al. (18); Johnson et al. (20); Jones et al. (21); Kaufer et al. (28)]	41.2%
Upper middle-income countries		
Brazil	2 [Fumis et al. (17); Midega et al. (22)]	11.8%
Lebanon	1 [Hajj et al. (19)]	5.9%
Lower middle-income countries		
India	2 [Atri et al. (13); Bhatt et al. (14)]	11.8%
Study design		
Cluster-randomized trial	1 [Curtis et al. (16) (USA)]	5.9%
Before–after interventional trials	2 [Johnson et al. (20) (USA); Amass et al. (12) (USA and Italy)]	11.8%
Cohort studies	2 [Gerstel et al. (18) (USA); Schwarzkopf et al. (24) (Germany)]	11.8%
Observational survey-based: Prospective	6 [Heyland et al. (26) (Canada); Min et al. (23) (South Korea); Nayfeh et al. (27) (Canada); Fumis et al. (17) (Brazil); Atri et al. (13) (India); Bhatt et al. (14) (India)]	35.3%
Observational survey-based: cross-sectional	2 [Hajj et al. (19) (Lebanon); Chuang et al. (15) (USA)]	11.8%
Observational survey-based: other (questionnaire/longitudinal)	2 [Kaufer et al. (28) (USA); Midega et al. (22) (Brazil)]	11.8%
Qualitative studies	1 [Al Mutair et al. (25) (Saudi Arabia)]	5.9%
Secondary/exploratory analyses	1 [Jones et al. (21) (USA)]	5.9%
Factors assessed		
Cultural factors only	3 [Heyland et al. (26); Al Mutair et al. (25); Nayfeh et al. (27)]	17.6%
Socioeconomic factors only	1 [Kaufer et al. (28)]	5.9%
Cultural and socioeconomic factors	13 [Amass et al. (12); Atri et al. (13); Hajj et al. (19); Bhatt et al. (14); Chuang et al. (15); Curtis et al. (16); Fumis et al. (17); Gerstel et al. (18); Johnson et al. (20); Jones et al. (21); Midega et al. (22); Min et al. (23); Schwarzkopf et al. (24)]	76.5%
Religion/Race/Ethnicity		
Religion		
Islam (Muslim)	1 [Al Mutair et al. (25)]	5.9%
Christianity	3 [Nayfeh et al. (27); Fumis et al. (17); Midega et al. (22)]	17.6%
Race/Ethnicity		
White	6 [Amass et al. (12); Chuang et al. (15); Curtis et al. (16); Gerstel et al. (18); Jones et al. (21); Nayfeh et al. (27)]	35.3%
Hispanic/Latino	2 [Chuang et al. (15); Jones et al. (21)]	11.8%
Black	1 [Chuang et al. (15)]	5.9%
Mediterranean	1 [Nayfeh et al. (27)]	5.9%
East Asian	1 [Nayfeh et al. (27)]	5.9%
Non-white (general)	1 [Kaufer et al. (28)]	5.9%
Other races	1 [Chuang et al. (15)]	5.9%
Unknown race	1 [Chuang et al. (15)]	5.9%
Tools used		
Interview	1 [Al Mutair et al. (25)]	5.9%
Quality of Dying and Death (QODD)	1 [Curtis et al. (16)]	5.9%
Family satisfaction with care (FS-ICU)	10 [Amass et al. (12); Atri et al. (13); Bhatt et al. (14); Chuang et al. (15); Gerstel et al., (18); Johnson et al. (20); Jones et al. (21); Kaufer et al. (28); Min et al. (23); Schwarzkopf et al. (24)]	58.8%
End-of-Life Satisfaction Survey	1 [Nayfeh et al. (27)]	5.9%
Modified Critical Care Family Needs Inventory (CCFNI)	1 [Midega et al. (22)]	5.9%
Hospital Anxiety and Depression Scale (HADS)	2 [Midega et al. (22); Amass et al. (12)]	11.8%
Modified Patient Judgement of Hospital Quality Questionnaire	1 [Heyland et al. (26)]	5.9%
Critical Care Family Satisfaction Survey (CCFSS)	1 [Hajj et al. (19)]	5.9%
Quality of Communication Questionnaire	1 [Chuang et al. (15)]	5.9%
End-of-Life Communication Tool	1 [Chuang et al. (15)]	5.9%
Impact of Event Scale—Revised (IES-R)	1 [Amass et al. (12)]	5.9%
Descriptive themes		
Communication and shared decision-making	8 [Curtis et al. (16) (USA); Johnson et al. (20) (USA); Jones et al. (21) (USA); Chuang et al. (15) (USA); Nayfeh et al. (27) (Canada); Atri et al. (13) (India); Bhatt et al. (14) (India); Schwarzkopf et al. (24) (Germany)]	38.1%
Emotional, psychological, and spiritual support	4 [Amass et al. (12) (USA and Italy); Al Mutair et al. (25) (Saudi Arabia); Fumis et al. (17) (Brazil); Hajj et al. (19) (Lebanon)]	19.04%
Cultural and religious sensitivity	4 [Al Mutair et al. (25) (Saudi Arabia); Nayfeh et al. (27) (Canada); Min et al. (23) (South Korea); Hajj et al. (19) (Lebanon)]	19.04%
ICU environment and institutional policies	4 [Gerstel et al. (18) (USA); Hajj et al. (19) (Lebanon); Fumis et al. (17) (Brazil); Johnson et al. (20) (USA)]	19.04%
Clinical care processes and outcomes	4 [Heyland et al. (26) (Canada); Curtis et al. (16) (USA); Amass et al. (12) (USA and Italy); Min et al. (23) (South Korea)]	19.04%

### 3.1 Country

The review included 17 studies in total, of which United States contributed the largest proportion (41.2%, seven studies), followed by Brazil, Canada, and India (11.8% each, two studies each), and single studies from Germany (5.9%), Lebanon (5.9%), Saudi Arabia (5.9%), and the Republic of Korea (5.9%), suggesting a gap in evidence from lower- and middle-income countries (LMIC).

### 3.2 Study design

When classified by study design, the largest category comprised observational, survey-based prospective studies (35.3%, six studies), conducted across multiple countries, including Canada, Brazil, South Korea, and India. Other survey-based designs, such as cross-sectional studies (11.8%, two studies) and questionnaire-based longitudinal observational studies (11.8%, two studies), were also frequently used. Cohort studies accounted for 11.8% (two studies), while before–after interventional trials also represented 11.8% (two studies), both mainly from the United States and Europe. Less frequently reported designs included a cluster-randomized trial (5.9%, one study), a qualitative phenomenological study (5.9%, one study) from Saudi Arabia, and a secondary exploratory analysis (5.9%, one study) from the United States. Overall, the evidence base is dominated by observational survey methodologies (58.9% combined), with fewer interventional and qualitative designs. This highlights a reliance on descriptive and exploratory approaches in the existing literature, with relatively limited experimental or randomized evidence to guide practice.

### 3.3 Religion/race/ethnicity

Of the 17 studies reviewed, only 9 (52.9%) explicitly reported the religion, race, or ethnicity of study populations. Religion was reported in four studies (23.5%), with Christianity being the most frequently cited (17.6%), followed by Islam (5.9%). Race and ethnicity were reported frequently, with White populations forming the majority (35.3%), while Hispanic/Latino populations were reported in 11.8% of studies. Other groups, including Black, Mediterranean, East Asian, non-white (general), other races, and unknown race, were each represented in a single study (5.9% each). Overall, these findings indicate limited reporting of cultural, racial, and religious characteristics, with a predominance of studies focusing on White and Christian populations. There is a clear need for studies involving populations of different religions, races, and ethnicities to better examine the influence of cultural and social determinants, highlighting a gap in the evidence.

### 3.4 Factors addressed

The studies were further classified according to whether they addressed cultural, socioeconomic, or both factors. Out of 17 studies, the majority (12–24) (*n* = 13, 76.5%) explored an interplay between cultural and socioeconomic influences on family satisfaction with ICU care. A smaller proportion of studies (25–27) (*n* = 3, 17.6%) focused exclusively on cultural factors, while only one study (28) (5.9%) examined socioeconomic factors in isolation. (Table 4).

### 3.5 Tools used

A diverse array of assessment tools was utilized across the 17 studies included in the review, with the Family Satisfaction in the Intensive Care Unit (FS-ICU) scale being the most widely employed, applied in 10 studies (58.8%) to evaluate family perspectives on ICU care and end-of-life care. Psychological distress was assessed in two studies (11.8%) using the Hospital Anxiety and Depression Scale (HADS), while bereavement stress was captured in one study using the Impact of Event Scale–Revised (IES-R). Other tools were applied in single studies (5.9% each), including the Quality of Dying and Death (QODD) questionnaire, the End-of-Life Satisfaction (EOLS) Survey, the Critical Care Family Needs Inventory (CCFNI), the Critical Care Family Satisfaction Survey (CCFSS), the Modified Patient Judgement of Hospital Quality (PJHQ) Questionnaire, the Quality of Communication (QOC) questionnaire, and a structured End-of-Life Communication tool. Additionally, one qualitative study utilized in-depth interviews.

### 3.6 Thematic description

Across the 17 studies reviewed, five major thematic domains emerged in relation to family satisfaction with ICU care.

#### 3.6.1 Shared decision-making and role of communication

This was addressed in eight studies (38.1%). These investigations consistently emphasized the role of honest, consistent, and empathetic communication in shaping family satisfaction. While Curtis et al. (16) (USA) and Johnson et al. (20) (USA) demonstrated that structured communication interventions improved family engagement in care decisions, studies such as Atri et al. (13) (India), Bhatt et al. (14) (India), Chuang et al. (15) (USA), Jones et al. (21) (USA), Nayfeh et al. (27) (Canada), and Schwarzkopf et al. (24) (Germany) highlighted that communication often outweighed clinical outcomes or ICU length of stay in determining overall satisfaction.

#### 3.6.2 Emotional, psychological, and spiritual support

Observed in four studies (19.04%), this finding highlighted the importance of addressing the family’s emotional and mental wellbeing alongside patient care. Amass et al. (12) (USA & Italy) demonstrated that structured rituals reduced post-traumatic stress in family caregivers, while Al Mutair et al. (25) (Saudi Arabia) and Hajj et al. (19) (Lebanon) highlighted the significance of spiritual and psychosocial support to the family members. Similarly, Fumis et al. (17) (Brazil) showed that open visitation policies improved family satisfaction by alleviating distress.

#### 3.6.3 Cultural and religious sensitivity

A comparable proportion of studies (19.04%) emphasized cultural and religious sensitivity as a determinant of family satisfaction. Al Mutair et al. (25) (Saudi Arabia) highlighted the centrality of religious practices in end-of-life care for Muslim families, while Nayfeh et al. (27) (Canada) revealed differences in experiences across White, Mediterranean, and East Asian families. Min et al. (23) (South Korea) described how Confucian traditions shaped prognosis communication, and Hajj et al. (19) (Lebanon) underscored the importance of culturally attuned information sharing.

#### 3.6.4 ICU environment and institutional policies

Reported in four studies (19.04%), Gerstel et al. (18) (USA) noted the impact of institutional culture on family perceptions, while Hajj et al. (19) (Lebanon) identified dissatisfaction with environmental comfort, particularly in waiting areas. Fumis et al. (17) (Brazil) highlighted the benefits of open visitation policies, and Johnson et al. (20) (USA) suggested that institutional reforms may be required to ensure significant improvements in family satisfaction.

#### 3.6.5 Clinical care processes and outcomes

Addressed in four studies (19.4%), findings consistently showed that families valued aspects such as symptom management, clear communication about prognosis, and alignment of care with patient wishes over purely clinical outcomes. Heyland et al. (26) (Canada) and Curtis et al. (16) (USA) demonstrated that caregiver satisfaction correlated more strongly with communication quality than with survival outcomes. Amass et al. (12) (USA and Italy) further revealed that structured interventions could enhance alignment of care with family expectations, while Min et al. (23) (South Korea) showed that extubation practices and discontinuation of life support in the ICU influenced satisfaction with both care and decision-making.

## 4 Discussion

This scoping review aimed to provide a comprehensive overview by mapping global evidence on the sociocultural and economic determinants that influence family satisfaction in the context of critically ill and palliative care in the ICU. The diverse factors influencing family satisfaction are often rooted in the sociocultural context of families, which shapes their involvement in clinical care, educational status, capacity to bear healthcare expenses, and the variability of healthcare infrastructure across different global settings (13, 19, 25). These findings underscore the need to recognize and address contextual factors and to tailor strategies to the distinct needs and expectations of family members within specific healthcare environments to ensure satisfaction. Key approaches involve simple yet impactful measures, such as addressing patients’ symptom relief, creating a safe ICU environment, implementing flexible visitation policies, ensuring honest and transparent communication with families to support shared or surrogate decision-making, providing emotional support, offering palliative care interventions, and attending to spiritual needs during palliative care.

This review noted that a significant proportion of the evidence originated from the United States and Canada, accounting for approximately 53% of the total. In contrast, there was a relative paucity of data from other regions of the world, despite the considerable variations in population demographics, race and ethnicity, sociocultural contexts, and economic factors across countries. However, the mapped evidence provides valuable insights into cultural and socioeconomic factors, as described below.

### 4.1 Cultural aspects

Cultural factors influencing family satisfaction were assessed in 94.1% of the included studies. A consistent finding across the included studies, mainly from North American countries, was that clear and frequent communication from the healthcare team, with due attention to the family’s spiritual and cultural needs, led to higher family satisfaction (12, 20, 21). This included providing families with complete information to support structured decision-making, providing emotional and psychological support to distressed and bereaved caregivers, and ensuring access to spiritual and chaplaincy services for patients at the end of life.

#### 4.1.1 Religious and spiritual beliefs surrounding health, illness, and death

Perceptions of families regarding health, illness, death, and grieving can be different depending on the religion and can influence family satisfaction (12, 25).

Irrespective of denomination (Catholic or Evangelical), Christians generally view illness and death not as an end, but as a passage to eternal life with God, depending on the good or bad deeds committed during one’s lifetime. Grieving families seek spiritual services, such as the presence of a pastor, to pray for the dying person and offer spiritual absolution. The sick person is anointed with holy oils and receives holy water, symbolizing the “body and blood of Christ” (29).

In Islam, illness is often viewed as a means of spiritual purification, and death is regarded as the beginning of the afterlife. In line with the “five pillars of Islam,” Muslims are encouraged to approach death with prayer, patience, recitation of the Qur’an, and reflection. Death is considered predestined, and families often strive to help their loved one recite the *Shahadatain*—the testimony of faith—at the end of life (25).

Similarly, Hindus see illness as a result of karma—actions from this life or past lives—and view death as a transition from one life to the next through reincarnation. Hindu families feel it is their sacred duty to assist the dying person at the end of life by reciting passages from sacred scriptures to provide spiritual comfort (30). Buddhists believe in the afterlife, with the ultimate goal of attaining “nirvana,” defined as freedom from the cycle of suffering and rebirth. Medications that can alter one’s state of mind at the end of life, such as narcotics, are often discouraged, as they may affect “one’s life transition and rebirth” (31). The available evidence predominantly reflects ICU practices and interventions from Christian-majority countries, with a noticeable focus on attending to the religious and spiritual demands of families within that context. There remains a substantial gap in research from other regions and non-Christian religious contexts, where caregiver perspectives remain underrepresented. This highlights an urgent need for prospective studies and randomized controlled trials in diverse healthcare environments to generate robust evidence to support best approaches aligned with caregivers’ local linguistic, religious, and spiritual needs.

This review also noted the positive impact of palliative care interventions directed at supporting end-of-life decision-making, resulting in higher family satisfaction (12, 13, 16, 18, 20, 27, 28). This highlights the value of integrating tailored end-of-life practices that cater to the diverse religious and spiritual requirements of families worldwide.

#### 4.1.2 Family role and dynamics

Patient’s ICU stay is a period of emotional upheaval for the families (17, 22). “Spiral of maintaining situational awareness, cognitive integration of facts, and difficult decision making” often requires collective effort from both families and the healthcare team. Interpersonal dynamics in caregiver families also play an important role in stress coping, grieving during bereavement, and end-of-life care situations (21, 28). The composition of families, interpersonal dynamics, and expectations surrounding filial responsibility differ markedly across global regions, shaped by distinct cultural, religious, and societal frameworks (32). While studies have shown a significant association between proactive participation of more educated families in shared decision-making for ICU care planning (28), the situation differs in LMIC. Studies from LMIC (13, 14) highlight how sociocultural contexts uniquely shape family satisfaction with ICU care. Families in many LMICs often come from rural backgrounds, have lower educational attainment, and live in larger household units, which influences their expectations and interactions with treating healthcare teams (13). These factors frequently shift the burden of surrogate decision-making onto clinicians, as relatives may feel less prepared or empowered to participate in complex medical decision-making (13, 14, 19). At the same time, strong extended-family networks can provide emotional support that enhances distress coping but may also create divergent opinions that lead to conflict (13). Together, these contextual elements underscore why family satisfaction dynamics in LMICs differ significantly from those in high-income settings.

Additionally, family support systems for patient care vary considerably across the globe depending on patient companion status (single/married//divorced), cohabitation (nuclear/joint family), and family dynamics related to filial responsibilities, distress coping, and shared decision-making. As most of the existing literature originates from Western ICU settings, its relevance in other diverse healthcare setups cannot be assumed. Existing evidence remains limited in scope, and future research should focus on examining how these contextual factors influence family satisfaction in multicultural settings.

#### 4.1.3 ICU environment and institutional policies

The ICU environment can be distressing for conscious patients and visiting family members (23–25). Noisy alarms from the monitors, the sight of multiple tubes and infusions, the physical and psychological distress of patients, and other grieving families nearby can all negatively impact a caregiver’s perception of ICU care, ultimately affecting their overall satisfaction.

However, several simple and targeted quality improvement initiatives can address these concerns and enhance satisfaction. These include ensuring optimal patient sensorium during visiting hours through appropriate sedation breaks; maintaining a calm and peaceful ICU environment during visitation periods; training bedside nurses to communicate effectively with families and address their queries; promptly attending to patients’ physical and psychological needs; providing comfortable and private spaces for grieving families; implementing flexible visitation hours to support family involvement; and creating a well-maintained and supportive ICU waiting room environment (13, 14, 17, 22, 25, 26). Additionally, timely multidisciplinary family meetings, early integration of end-of-life care services for terminally ill patients, and the presence of a dedicated medical social worker or psychologist to support distressed or grieving families are crucial steps toward delivering truly patient- and family-centered care (21, 22).

### 4.2 Socioeconomic aspects

#### 4.2.1 Healthcare expenditure and insurance coverage

Financial burden associated with ICU care, such as high treatment costs and long-term financial repercussions, can negatively affect family satisfaction, especially when there is a significant gap between medical costs and insurance coverage (33, 34). There is considerable variation in public awareness of healthcare expenditure and in the proportion of a nation’s gross domestic product (GDP) allocated to healthcare. These disparities can significantly impact out-of-pocket expenses (OOPEs) for caregivers and adversely affect overall family satisfaction with care (34). Caregivers may also face loss of income, as they are required to remain with patients in the hospital for prolonged periods, further increasing their financial burden.

This review identified significant gaps and a lack of evidence specifically examining the economic factors that influence family satisfaction with ICU care, thereby overlooking several important dimensions. Family satisfaction with healthcare can vary significantly in countries such as India and Lebanon (with healthcare spending of 3.3 and 6% of gross domestic product, respectively, and less developed government or private insurance coverage supplementation) as compared to nations such as the United States and Canada (with higher health expenditure—approximately 17 and 12.3% of GDP, respectively—well-supported universal or mandated coverage systems) (35). In settings with low public awareness of healthcare costs and limited government-sponsored health coverage, families often face highly variable and sometimes catastrophic OOPEs. The wide rich–poor divide in LMICs further amplifies these disparities, leaving vulnerable families particularly exposed during episodes of critical illness. Strengthening government-backed comprehensive health coverage and improving public understanding of financial risk protection mechanisms are, therefore, crucial steps in the LMIC context. Such measures can substantially enhance family satisfaction by reducing financial distress during ICU admissions.

There is an urgent necessity for high-quality research that specifically explores how financial burden influences caregiver satisfaction with ICU care, especially in LMICs. Such evidence is essential for policymakers at both national and international levels.

#### 4.2.2 Educational aspects

Active family participation in ICU decision-making depends on various factors such as caregivers’ educational status, understanding of the illness, and the number of decision-makers in the family. This review noted that young, educated relatives of patients often require clear, honest, and frequent communication from the healthcare team to support shared decision-making (19, 26). In contrast, less educated families often prefer the treating team to make surrogate decisions on their behalf (13, 14). Novel interventions, such as educational initiatives for the families of ICU patients regarding the ICU environment, routine ICU procedures, the multidisciplinary team approach for patient care, infection control, end-of-life care, and common issues such as pain and sedation, have shown promise in reducing anxiety and improving family satisfaction (36). However, more studies are required to explore approaches that improve family satisfaction across diverse educational backgrounds.

### 4.3 Strengths and limitations

This scoping review’s strength lies in its detailed and comprehensive approach. It uniquely considers the geographical, economic, sociocultural, and religious contexts in which care is delivered. The facilitation of respectful and meaningful terminal care through palliative, comfort-based services in ICUs has been gaining global attention. This review represents one of the first comprehensive efforts to evaluate how end-of-life care practices affect family satisfaction within varying sociocultural and spiritual frameworks. A rigorous search strategy, using carefully constructed search terms across five major databases, further broadened the scope and depth of this review. This review also had several limitations. The literature review revealed that a significant proportion of the evidence originated in North America, potentially limiting the generalizability of the findings to other regions. Additionally, the review was limited to studies published in English, potentially overlooking relevant studies in other languages. The included studies were heterogeneous, encompassing diverse methodologies, from randomized controlled trials and observational studies to qualitative research, and were subject to inherent methodological limitations. Measures of family satisfaction with ICU care varied considerably, ranging from objective tools such as the FS-ICU, CCFNI, and IES-R scales to qualitative approaches, such as family interviews. Finally, despite comprehensive efforts to develop an inclusive search strategy, it is possible that some pertinent studies were unintentionally overlooked.

### 4.4 Policy and practice implications

This scoping review provides insights to guide the development of future practice and policy measures at multiple levels, as outlined below.

- At the individual level:

Clinician training to recognize diverse religious beliefs, cultural norms, and family expectations surrounding illness and death.

Use of culturally appropriate language and communication strategies during family meetings.

Implementation of structured assessment frameworks to routinely assess sociocultural and economic factors that affect families’ perceptions and satisfaction with ICU care (such as religious needs, healthcare cost constraints, decision-making capacity, and stress-coping mechanisms).

Tailoring decision-making discussions based on the family’s preferred level of involvement, filial responsibilities, literacy, and stress-coping abilities.

Provision of social work consultations and financial counseling for psychological and economic support.

- At the institutional level:

Implementation of flexible ICU visitation policies that align with patient and family needs.

Creation of a supportive ICU environment by providing waiting areas with amenities, private rooms for family discussions, and quiet spaces for prayer.

Establishment of feedback systems for families to report concerns, dissatisfaction, or cultural mismatches and use of these data for continuous quality improvement in family-centered ICU care.

Incorporation of communication skills training and cultural competence as a part of capacity-building initiatives.

- At the national policy level:

Strengthening national healthcare coverage through policies that guarantee access to essential medical services, thereby reducing financial barriers to ICU care.

## 5 Conclusion

Holistic, patient- and family-centered care in the ICU has become an urgent priority worldwide. When evaluating caregiver satisfaction in the ICU, it is essential to consider the influence of diverse cultural, religious, and socioeconomic contexts. However, there remains a critical lack of evidence from LMICs, limiting the ability to fully inform and guide practice in these settings.

## Data Availability

No new data were generated or analyzed in this study. All original research articles included in the scoping review are publicly available through scientific databases. The search strategy used for study identification is provided in the Supplementary Appendix. The dataset is available with corresponding author upon reasonable request.
