# Supplementary material for: Cultural and socioeconomic determinants of family satisfaction with ICU care across the globe: a scoping review
Source: Front Med (Lausanne). 2026 Jan 15;12:1700383. doi: 10.3389/fmed.2025.1700383 (PMC12852357; doi:10.3389/fmed.2025.1700383)
Supplement: Supplementary file 1 [file Table_1.DOCX]

**Appendix 1**

Additional Search Strategy Segments

The following broader thematic strings supplemented the core strategy to ensure comprehensiveness:

- Socioeconomic factors:
  "Socioeconomic Factors"[MeSH] OR "Poverty"[MeSH] OR "Health Care Costs"[MeSH] OR "Income"[MeSH] OR "education"[Title/Abstract]
- Cultural influences:
  "Communication Barriers"[MeSH] OR "Social Determinants of Health"[MeSH] OR "Attitude to Health"[MeSH] OR "cultural factors"[Title/Abstract]
- Family and ICU population:
  "Family"[MeSH] OR "Caregivers"[MeSH] OR "patient relatives"[Title/Abstract]
- ICU settings:
  "Critical Care"[MeSH] OR "Intensive Care Units"[MeSH] OR ICU[Title/Abstract] OR "Ventilator care"[Title/Abstract]

**Search strings for databases**

| Database | Search string |
| --- | --- |
| PubMed | ("Intensive Care Units"[MeSH] OR "Critical Care"[MeSH] OR ICU[Title/Abstract] OR "intensive care"[Title/Abstract]) AND ("Cultural Characteristics"[MeSH] OR "Culture"[MeSH] OR "Cross-Cultural Comparison"[MeSH] OR "Ethnic Groups"[MeSH] OR "cultural factors"[Title/Abstract] OR "cultural background"[Title/Abstract] OR "ethnicity"[Title/Abstract] OR "cross-cultural"[Title/Abstract] OR "religion"[Title/Abstract] OR "spiritual support"[Title/Abstract]) AND ("Socioeconomic Factors"[MeSH] OR "Income"[MeSH] OR "Educational Status"[MeSH] OR "Occupational Status"[Title/Abstract] OR "socioeconomic status"[Title/Abstract] OR "income"[Title/Abstract] OR "education"[Title/Abstract] OR "employment"[Title/Abstract] OR "economic status"[Title/Abstract] OR "social class"[Title/Abstract]) AND ("Family Satisfaction"[Title/Abstract] OR "Family"[MeSH] OR "Patient Satisfaction"[MeSH] OR "Family Relations"[MeSH] OR "family perception"[Title/Abstract] OR "family experience"[Title/Abstract] OR "family needs"[Title/Abstract] OR "family perspective"[Title/Abstract] OR "FS-ICU 24R"[Title/Abstract]) |
| Embase | ('intensive care unit'/exp OR 'critical care'/exp OR 'icu':ti,ab,kw OR 'intensive care':ti,ab,kw OR 'critical care':ti,ab,kw) AND ('cultural characteristics'/exp OR 'culture'/exp OR 'cross-cultural comparison'/exp OR 'ethnic groups':ti,ab,kw OR 'cultural factors':ti,ab,kw OR 'cultural background':ti,ab,kw OR 'ethnicity':ti,ab,kw OR 'cross-cultural':ti,ab,kw OR 'religion':ti,ab,kw OR 'spiritual support':ti,ab,kw) AND ('socioeconomic factors'/exp OR 'income'/exp OR 'education status' OR 'occupational status':ti,ab,kw OR 'socioeconomic status':ti,ab,kw OR 'income':ti,ab,kw OR 'education':ti,ab,kw OR 'employment':ti,ab,kw OR 'economic status':ti,ab,kw OR 'social class':ti,ab,kw) AND ('family'/exp OR 'patient satisfaction'/exp OR 'family satisfaction':ti,ab,kw OR 'family perception':ti,ab,kw OR 'family experience':ti,ab,kw OR 'family needs':ti,ab,kw OR 'family perspective':ti,ab,kw OR 'fs-icu 24r':ti,ab,kw) |
| Web of Science | TS=("Intensive Care Units" OR "Critical Care" OR ICU OR "intensive care" OR "critical care") AND TS=("Cultural Characteristics" OR "Culture" OR "Cross-Cultural Comparison" OR "ethnic groups" OR "cultural factors" OR "cultural background" OR ethnicity OR "cross-cultural" OR religion OR "spiritual support") AND TS=("Socioeconomic Factors" OR "Income" OR "Educational Status" OR "occupational status" OR "socioeconomic status" OR income OR education OR employment OR "economic status" OR "social class") AND TS=("Family Satisfaction" OR "Family" OR "Patient Satisfaction" OR "Family Relations" OR "family perception" OR "family experience" OR "family needs" OR "family perspective" OR "FS-ICU 24R") |
| Scopus | TITLE-ABS-KEY ("intensive care unit" OR "critical care" OR ICU OR "intensive care" OR "critical care") AND TITLE-ABS-KEY ("cultural characteristics" OR "culture" OR "cross-cultural comparison" OR "ethnic groups" OR "cultural factors" OR "cultural background" OR ethnicity OR "cross-cultural" OR religion OR "spiritual support") AND TITLE-ABS-KEY ("socioeconomic factors" OR "income" OR "education status" OR "occupational status" OR "socioeconomic status" OR "income" OR "education" OR "employment" OR "economic status" OR "social class") AND TITLE-ABS-KEY ("family satisfaction" OR "family" OR "patient satisfaction" OR "family relations" OR "family perception" OR "family experience" OR "family needs" OR "family perspective" OR "FS-ICU 24R") |
| Cochrane | ("intensive care" OR "critical care" OR ICU) in Title Abstract Keyword AND ("cultural characteristics" OR "culture" OR "cross-cultural" OR "ethnicity" OR "religion" OR "spiritual support") AND ("socioeconomic factors" OR "income" OR "education" OR "employment" OR "social class") AND ("family satisfaction" OR "patient satisfaction" OR "family experience" OR "family needs") in Title Abstract Keyword |
